# Objective Assessment of Media Screens Effect on Physical Activity in Preschool Children

**DOI:** 10.1109/access.2026.3729105

**Published:** 2026-09-01

**Authors:** MD BILLAL HOSSAIN, STEVEN HOLIDAY, CHUONG BUI, MATTHEW CRIBBET, SUSAN W. WHITE, YU GAN, EDWARD SAZONOV

**Affiliations:** 1 Department of Electrical and Computer Engineering, The University of Alabama, Tuscaloosa, AL 35487, USA; 2 Department of Advertising and Public Relations, The University of Alabama, Tuscaloosa, AL 35487, USA; 3 Alabama Life Research Institute, The University of Alabama, Tuscaloosa, AL 35487, USA; 4 Department of Psychology, The University of Alabama, Tuscaloosa, AL 35487, USA; 5 Fischell Department of Bioengineering, University of Maryland, College Park, MD 20742, USA

**Keywords:** Activity intensity, preschool children, screen time, wearable sensor

## Abstract

Media screen time is linked to physical and psychosocial health outcomes in preschool children (≤5 years), but evidence largely relies on parent reports or a single device type. This study proposes the use of a novel sensor, Screen Time Tracker (STT), to perform an objective assessment of the effect of screen time on physical activity in preschool children under free-living conditions. Egocentric images (10-s intervals) and triaxial accelerometer data (128 Hz) were collected from 25 children aged 3–5 years using the STT device. Accelerometer data were processed to estimate activity Counts Per Minute (CPM). A deep learning model was used to detect multiple types of active screens in images and compute Screen Counts per Minute (SCM; range, 0–6). On average, children exhibited lower CPM during screen interaction (1181 for SCM > 0) than during screen-free minutes (1628 for SCM = 0). Among screen interaction minutes, CPM decreased progressively with increasing SCM (from 1629 at SCM = 1 to 132 at SCM = 6). A multilevel negative binomial model with a log link, accounting for minutes nested within days and days nested within children, showed that higher SCM above a child’s own daily mean was significantly associated with lower activity counts in a typical child on a typical day (b = −0.228, p < .001, exp[b] = 0.796). Thus, for each one-count increase in SCM above a child’s daily mean, expected CPM decreased by approximately 20.4%. Furthermore, higher sustained screen interaction minutes were associated with lower physical activity. Children who continuously engaged with screens for 2 minutes showed mean CPMs of 1421, while continuous engagement for 15 minutes or more had a mean CPM of 693. Multi-level analysis showed that longer SSIM bouts were associated with lower mean CPM (b = −46.677, p = .005). This proof-of-concept study objectively shows that increased screen engagement is associated with lower physical activity intensity in preschool children. These findings provide useful evidence for epidemiologists, pediatricians, and behavioral scientists studying the health effects of media screen use in children.

## I. INTRODUCTION

The impact of media screen time (ST; i.e., time spent on computers, laptops, tablets, cell phones, televisions, and so on) on physical activity, energy intake, mental health (anxiety, depression, and internalizing problems, behavioral problems, self-esteem), sleep disruption, cognitive development, and social interaction has gained significant attention in recent years. The research has focused particularly on children and adolescents (aged birth to 18–19 y.o.) [1], [2]; however, preschool children (aged 2.5–5 y.o.) are particularly vulnerable, as early habits form the foundation for long-term developmental trajectories.

Studies in children and adolescents have shown that more than 2 hours of daily Screen Time (ST) is associated with decreased cardiorespiratory and lower aerobic fitness [3]. According to the American Academy of Child and Adolescent Psychiatry, children ages 8–18 in the United States spend 7.5 hours daily on average in front of a media screen [4]. A parent report-based study shows that children under 5 years have 26 hours per week of screen time, which exceeds the recommended time [5]. Excessive screen time encourages prolonged sitting or inactivity, which could lead to sedentary behavior [4], [6], [7], [8] and, in turn, affect academic performance [9]. Some studies have found a correlation between screen media exposure, obesity [10], [11], and body mass index [12]. Some studies have found a negative relationship between screen time and Physical Activity (PA) intensity and duration [2], [13]. A longitudinal study based on proxy respondents and self-report methods found that excessive screen time at 2 years of age led to less outdoor playing time 8 months later [14]. In the same study, analysis of the National Health and Nutritional Examination Survey study data on more than 1,000 U.S. children aged 6–15 showed that higher TV viewing was associated with lower physical strength, irrespective of physical activity [14]. A review study showed that screens in the home environment are positively related to sedentary behavior [15]. However, no existing method objectively quantifies real-world screen exposure and physical activity simultaneously in preschoolers, particularly in natural, unsupervised daily environments.

Epidemiological studies have shown inconsistent links between screen time and reduced PA for preschoolers cross-sectionally and longitudinally. One of the reasons is the difficulty in validly measuring concurrent screen media exposure time and PA [11]. Various methods have been documented for tracking screen use in preschool children. A comprehensive review of screen media use tracking tools is given in [16]. The most popular method is parent reporting [6], [10], [16]. However, the method is not reliable and is prone to overestimation and underestimation errors reported by several research articles [17], [18], [19], [20]. Some researchers have used direct or indirect observation methods, which have limited scope, are time-consuming, and are subject to observation bias [21].

However, self-report methods [12], [13], [14], [21] are subjective and can lead to biases and inaccuracies, particularly in preschool children, due to memory lapses and judgment errors [16], [22]. In fact, evidence from a systematic review shows that children younger than 5 years old cannot validly or reliably self-report health outcomes [23]. Other researchers used technology-based tracking methods (e.g., tracking software, video recording, eye tracking, and color sensors), albeit within a limited scope. For example, in [17], the researcher used Chronicle tracking software for ST detection in the home environment, which runs only on Android and iOS-based operating systems. Another limitation is the inability of passive sensing to determine who is using the device. Although the study attempted to exclude shared-device data, this approach is not practical for larger-scale free-living conditions. Studies in [24] and [25] employed the video recording method, which is restricted to TV viewing in the home environment, excluding other screen types and out-of-home contexts. Although eye tracking can provide direct information about children’s visual attention, its use in preschool children remains challenging due to calibration difficulties, participant compliance, device slippage, movement, and data-quality issues [26], [27], [28]. These challenges make eye tracking difficult to implement at scale for continuous free-living ST assessment. Based on a systematic review [16], most objective assessments of preschool children’s ST have been conducted in home or controlled environments and focused on specific screen types.

To address these limitations, we introduce the Screen Time Tracker (STT) [29], [30] — a wearable system enabling egocentric multi-screen detection across TVs, smartphones, tablets, computers, and in-vehicle displays; synchronized accelerometry-derived Counts Per Minute (CPM) measurement equivalent to ActiGraph for assessing movement levels and activity intensity [31], [32], [33], [34]; and free-living continuous monitoring.

To our knowledge, this is the first system capable of continuous, objective, multimodal measurement of screen exposure and physical activity in preschool-aged children under free-living conditions. In this proof-of-concept study, we used the STT to investigate the association of objectively measured screen-interaction frequency and duration with PA intensity in preschool-aged children under free-living conditions. Specifically, we examined: (1) a moment-to-moment relationship between screen detection frequency and activity intensity, and (2) whether longer consecutive minutes of screen exposure bouts corresponds to greater activity suppression.

The major contributions of this research are:

- A novel wearable sensing framework combining egocentric imaging and accelerometry to capture real-world screen exposure and physical activity.
- A non-intrusive method for objective screen exposure detection not limited to a specific screen type, overcoming the limitations of parent-reports, home-only monitoring, or device-specific logging.
- Introduction of Screen Counts per Minute (SCM) and Sustained Screen Interaction Minutes (SSIM) as quantitative digital markers of screen engagement.
- Initial proof-of-concept evidence, based on the proposed digital markers, that increasing screen interaction frequency and duration in free-living preschoolers is associated with significant decreases in physical activity.

The following sections provide a detailed description of the methodology, results, and conclusions of our findings.

## II. MATERIALS AND METHODS

### A. WEARABLE SENSOR SYSTEM

**FIGURE 1.**
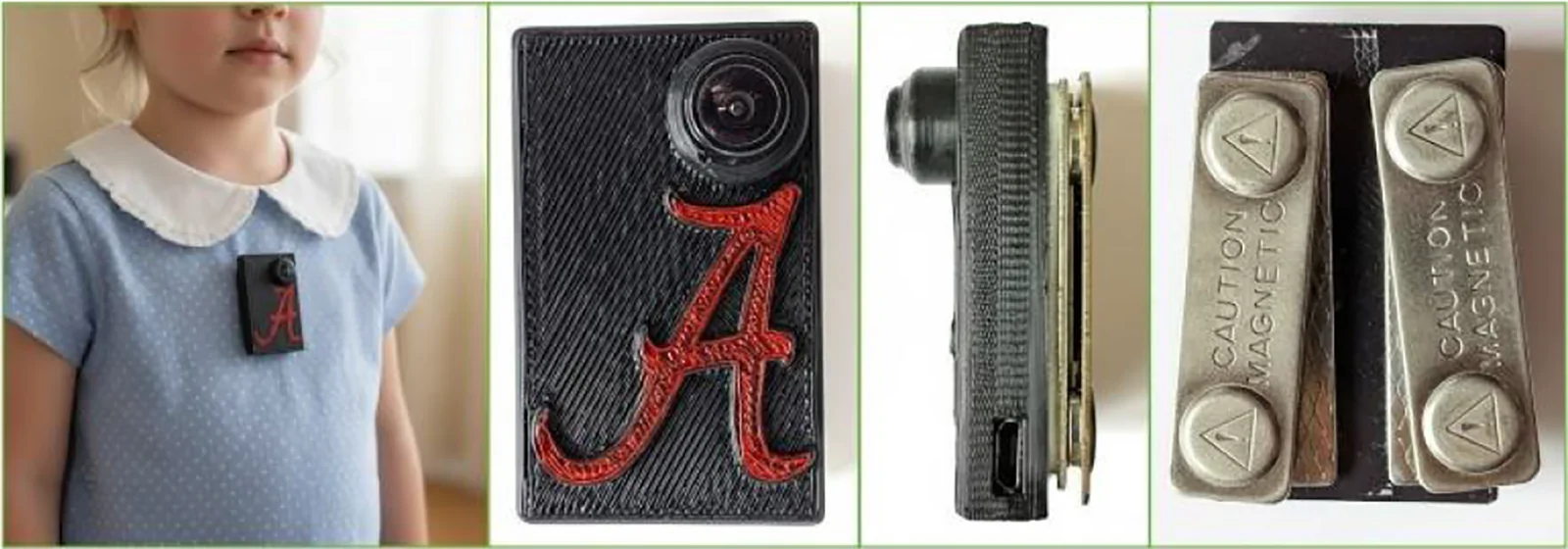
Screen time tracker device. The left panel shows an AI-generated [37] illustration of a child wearing the STT device. The remaining panels show the device’s front, side, and bottom views.

We collected data using an egocentric camera called the STT [29], [35]. The STT contains a compact 5-megapixel camera with a 120-degree wide-angle resolution of 2592 × 1944 pixels. The image capture time is stored in the filename, with images captured at 10-second intervals. In addition, STT included a low-power 3D accelerometer (ADXL362 from Analog Devices, Norwood, MA, USA). The accelerometer was configured with 128 Hz samples per second and a dynamic range of ±2 g. The camera was mounted on clothing using a magnetic clip, as shown in Fig. 1, and had a battery life exceeding 48 hours. With dimensions of 54 mm × 35 mm × 12 mm, the STT was compact, occupying only 30% of the size of commercial cameras [36].

### B. DATA COLLECTION PROCESS AND PROTOCOLS

Preschool children aged 3 to 5 years were recruited for participation in the study following written informed parental consent. The study protocol received approval from the Institutional Review Board (Protocol # 20-07-3713) at the University of Alabama. Data were collected from 34 participants, each of whom was instructed to wear the device during waking hours over one or two calendar days to capture free-living activities, while maintaining normal routines and removing the device as needed for privacy, sleep, naps, or bathing. However, due to device malfunctions, usable data were obtained from only 25 participants (12 males, 13 females, average (std) age 4.67 (1.00)). Data collection comprised 33 participant-days (17 participants monitored for one day; 8 for two days).

### C. OBJECTIVE ASSESSMENT OF ST EFFECT ON PA

**FIGURE 2.**
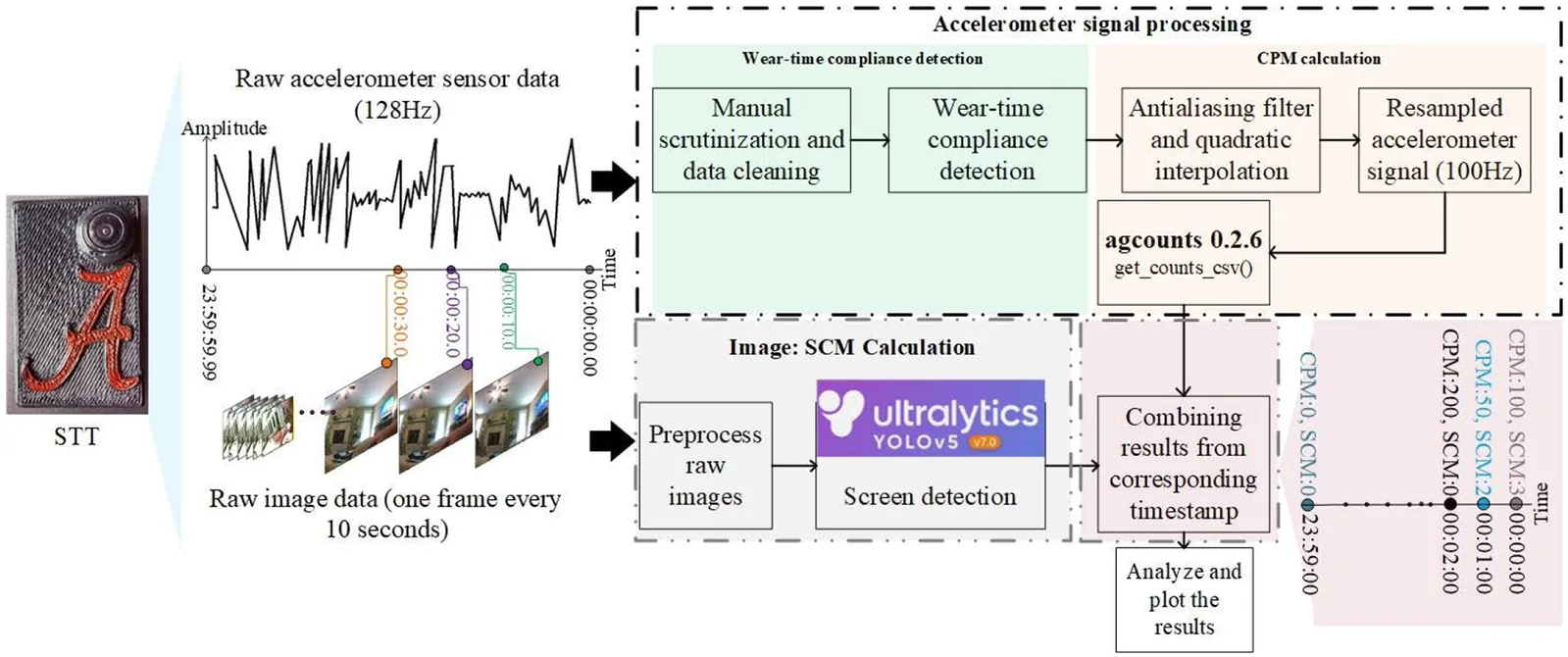
Overview of the proposed objective assessment of ST effect on PA.

The overview of the proposed objective assessment of ST effect on PA is illustrated in Fig. 2. To perform the objective assessment, we introduced two digital markers of screen engagement: SCM and SSIM. SCM denotes the number of screen detections within each minute (0–6, given 10-s image sampling). SCM = 0 indicates no screen appearances; higher values signify more frequent and sustained screen exposure within that minute.

Each minute with available STT data was classified either as Screen-Free Minute (SFM; SCM = 0) or Screen-Interaction Minute (SIM; SCM > 0). Sustained Screen-Interaction Minutes (SSIM) were defined as sequences of ≥2 consecutive SIM minutes. For example, a 3-minute SSIM indicates screen exposure in each of exactly three consecutive minutes, with longer SSIM values representing longer consecutive minutes of screen engagement; the 15-minute category includes 15 or more consecutive minutes.

Accelerometer signals are processed to compute CPM, while egocentric images are processed in parallel to detect active screens and estimate SCM. The CPM and SCM metrics are then aligned using timestamps. To ensure consistency, all CPM values were rounded to the nearest integer. Each processing step was validated individually (see Section III) prior to integrating them into the complete workflow. The following subsections provide a detailed description of each processing step and validation process.

### D. ACCELEROMETER SIGNAL PROCESSING

Accelerometer signal processing consisted of two major steps: wear-time compliance detection and CPM calculation. The following subsections describe these processing steps in detail.

#### 1) WEAR-TIME COMPLIANCE DETECTION

A manual scrutiny of the collected data was performed first by plotting the accelerometer signals to identify missing sensor data, stationary signal patterns, and possible non-wear intervals. For each participant-day, on-time was defined as the total duration that the device remained unplugged from the charger and included both normal-wear and non-wear time [38]. Manual review of the sensor and image data suggested that most non-wear time was non-wear-stationary. Other non-wear subtypes defined in [38], namely non-compliant-wear and non-wear-carried, were rare in this dataset; therefore, we treated non-wear time as non-wear-stationary throughout. Non-wear intervals were identified through manual annotation of chronologically ordered timestamped image sequences, verified against full-size images and accelerometer signal plots, where no scene changes and a stationary signal confirmed the device was not being worn. All remaining on-time intervals were classified as normal-wear.

After manual annotation, a threshold-based algorithm then classified each 1-s epoch as normal-wear or non-wear by computing the vector magnitude of the triaxial accelerometer signal (Eq. 1), smoothing the mean-centered RMS using a 180-s fastsmooth filter [39] (type 3), and epochs exceeding the threshold were labeled as normal-wear; otherwise, as non-wear (Eqs. 2–4).


(1) $$m(t)=\sqrt{a_{x}(t{)}^{2}+a_{y}(t{)}^{2}+a_{z}(t{)}^{2}}$$

(2) $$RMS_{k}=\sqrt{\frac{1}{N}\overset{N}{\underset{i=1}{\sum }}{\left(m_{k,i}-{\overset{‾}{m}}_{k}\right)}^{2}}$$

(3) $$\tilde{RMS_{k}}=\text{fastsmooth}\left(RMS_{k}\right)$$

(4) $${\overset{ˆ}{y}}_{k}=\left\{\begin{array}{ll} \text{normal}-\text{wear}, & \tilde{RMS_{k}}>T\\ \text{non}-\text{wear,} & \tilde{RMS_{k}}\leq T \end{array}\right.$$

The threshold *T* = 6 was selected using leave-one-subject-out validation, with normal-wear treated as the positive class and non-wear treated as the negative class. The optimal value was chosen based on the highest F1 score. After threshold-based detection, only algorithm-detected normal-wear intervals were retained; algorithm-detected non-wear intervals and their corresponding images were excluded from further analysis.

#### 2) CPM CALCULATION

CPM was estimated from the retained normal-wear accelerometer data. In this study, CPM was computed using the open-source Python package agcounts version 0.2.6 [40]. This package was developed by ActiGraph (Pensacola, FL, USA) [41] and computes ActiGraph-compatible activity counts from raw accelerometer signals. The 128 Hz accelerometer signal was downsampled to 100 Hz for compatibility with the agcounts package. This was achieved by applying a 4th-order Butterworth low-pass filter with a cutoff frequency of 50 Hz to prevent aliasing, followed by quadratic interpolation. The activity counts in CPM were calculated using the get_counts_csv() method provided by agcounts version 0.2.6, with epoch = 60 s and freq = 100 Hz as input arguments. A full-day accelerometer signal with timestamps in the format recommended by the package was used as input and only counts within the normal-wear intervals were retained for analysis. The package combines all three accelerometer axes to compute activity counts. No additional calibration was applied because agcounts computes ActiGraph-compatible counts directly from raw accelerometer signals.

**FIGURE 3.**
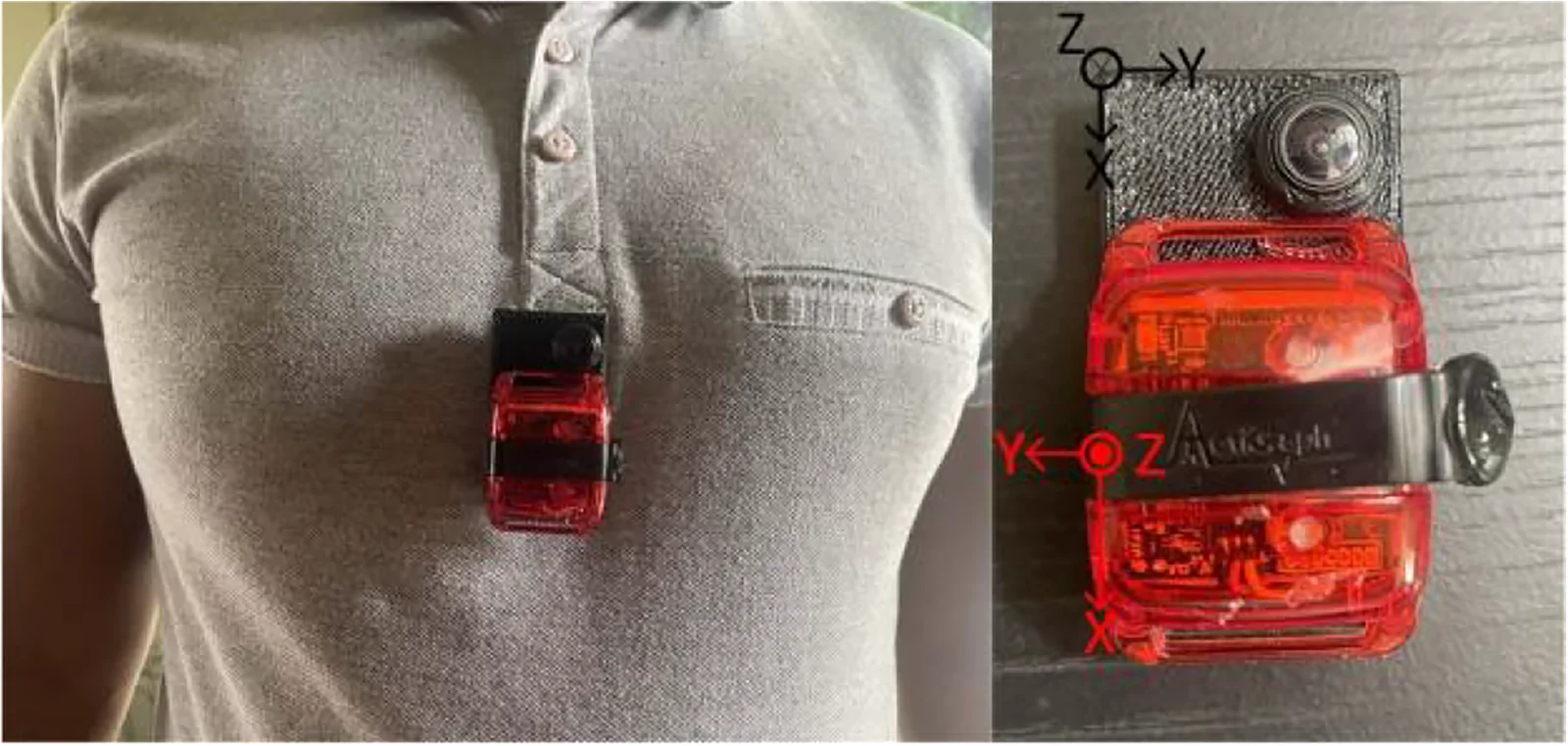
STT and wGT3X-BT taped together for validation of CPM calculation.

The CPM is influenced by device-specific factors, including differences in accelerometer models, placement, and orientation [42]. Therefore, ActiGraph devices were used as the reference for CPM comparison because they are widely used in PA research and have been utilized in more than 20,000 scientific publications by the end of 2021 [42]. To validate activity count accuracy, STT CPM values were compared with ActiGraph wGT3X-BT [43] CPM values. We followed the validation approach described in [44]. The two devices were securely attached using strong double-sided tape (Fig. 3). Two adult volunteers wore the devices on their chest—positioned identically to children’s study—while performing free-living daily activities totaling 743 minutes of recording. CPM values were calculated from the raw accelerometer signals of the STT and wGT3X-BT devices using the agcounts package. Activity levels were classified using adult-based CPM thresholds [45]. Agreement between the two devices was assessed using scatter, Bland–Altman, Pearson correlation, Lin’s concordance correlation coefficient plots, and numerical metrics, including average deviation, mean absolute error, and root mean square error.

### E. IMAGE PROCESSING, SCREEN DETECTION, AND SCM CALCULATION

The image processing pipeline used to calculate SCM is shown in Fig. 2. Raw egocentric images were first preprocessed using barrel distortion correction [46], and low-light enhancement with MBLLEN [47]. Egocentric images often feature dynamic, cluttered backgrounds due to the nature of first-person perspectives [48], making traditional image classification models like those trained on ImageNet-21K [49] unsuitable. Therefore, we employed an object detection approach, YOLOv5l6, that has shown superior performance on egocentric images in prior work [50].

The YOLOv5l6 model, pretrained on the MS COCO dataset [51], was fine-tuned to detect active (powered-on) screens using data from two adult studies. Study-1 [52] was conducted at the University of Alabama, while Study-2 was conducted at Boston University (MA) and Miriam Hospital (RI). Institutional Review Board approval was obtained for each study prior to data collection. Both studies employed the AIM-2 wearable device, which shares the same camera module as the STT device, but was placed on a different body position (was worn on the eyeglass frame at head level). The combined dataset included 66 participants (16,801 screen images and 4,405 negative examples), with 30 participants from Study-1 and 36 from Study-2. Screen images were defined as images containing any visible powered-on display, such as televisions, smartphones, tablets, or computer monitors. A subset of images from each participant was manually annotated for screen presence, sampled primarily around food-intake periods, and supplemented with additional screen-only instances. We randomly selected images from 3 participants (579 screen images and 130 negative examples from Study-2) collected in free-living environments for validation. Our dataset encompassed various media screens, including TVs, cell phones, smartphones, tablets, computers, laptops, and car entertainment systems. We fine-tuned the YOLOv5l6 model for up to 150 epochs with an input image size of 640 × 640 pixels. Various image augmentation techniques, including flipping (vertical and horizontal), mosaic, mix-up, copy-paste, rotation, translation, rescaling, and shearing, were applied to enhance the model’s generalization capabilities. Early stopping was utilized to mitigate overfitting. The confidence threshold for screen detection was determined using a receiver operating characteristic curve to minimize false SIM detections. On the validation datasets from Study-1 and Study-2, the fine-tuned model demonstrated strong object detection performance, achieving the following metrics [51]: precision (0.95), recall (0.85), mAP@0.5 (0.94), and mAP@0.5–0.95 (0.74).

**FIGURE 4.**
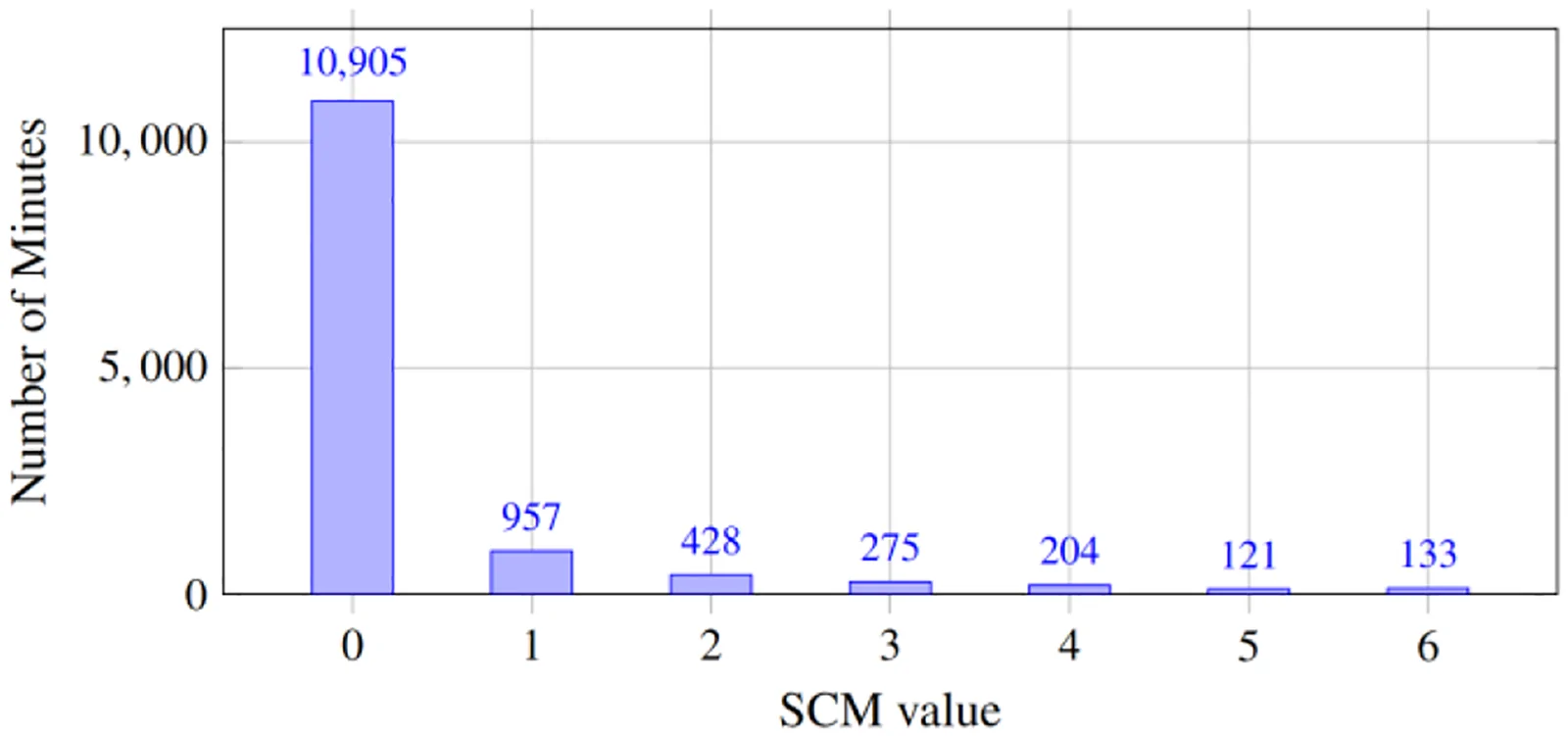
Histogram of SCM.

**FIGURE 5.**
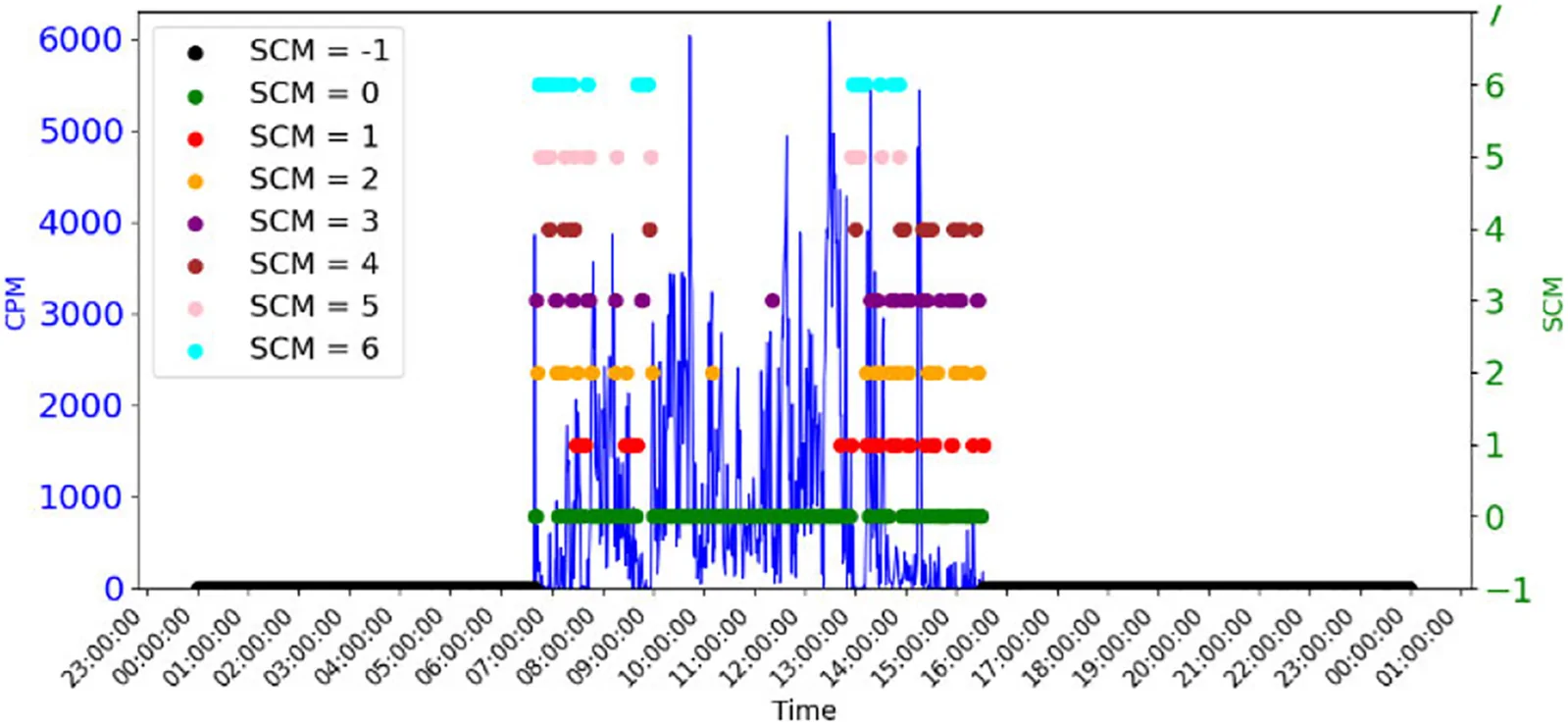
Sample CPM and SCM plot for participant ID: STT235177 for one complete day.

The YOLOv5l6 screen detection model was then applied to the algorithm-detected normal-wear interval images (13,023 minutes or 217.05 h) from the children’s study. An image was classified as a screen image if at least one powered-on screen was detected. SCM was then computed as the number of screen images detected within each minute. The model identified 10,905 minutes as SFM (SCM = 0) and 2,118 minutes as SIM (SCM>0). Fig. 4 illustrates the distribution of SCM detections, while Fig. 5 presents an example of CPM and SCM values over one day for a single participant. The SCM value of −1 indicates no device use (non-compliance). The participant was active from around 7:00 AM until 4:00 PM, with varying SCM values throughout the active period.

## III. RESULTS AND DISCUSSIONS

The Results and Discussions section is organized into four subsections: validation of CPM calculation, validation of screen image detection and SCM Calculation, validation of wear-time compliance detection, and ST Effect on PA intensity.

### A. VALIDATION OF CPM CALCULATION

**FIGURE 6.**
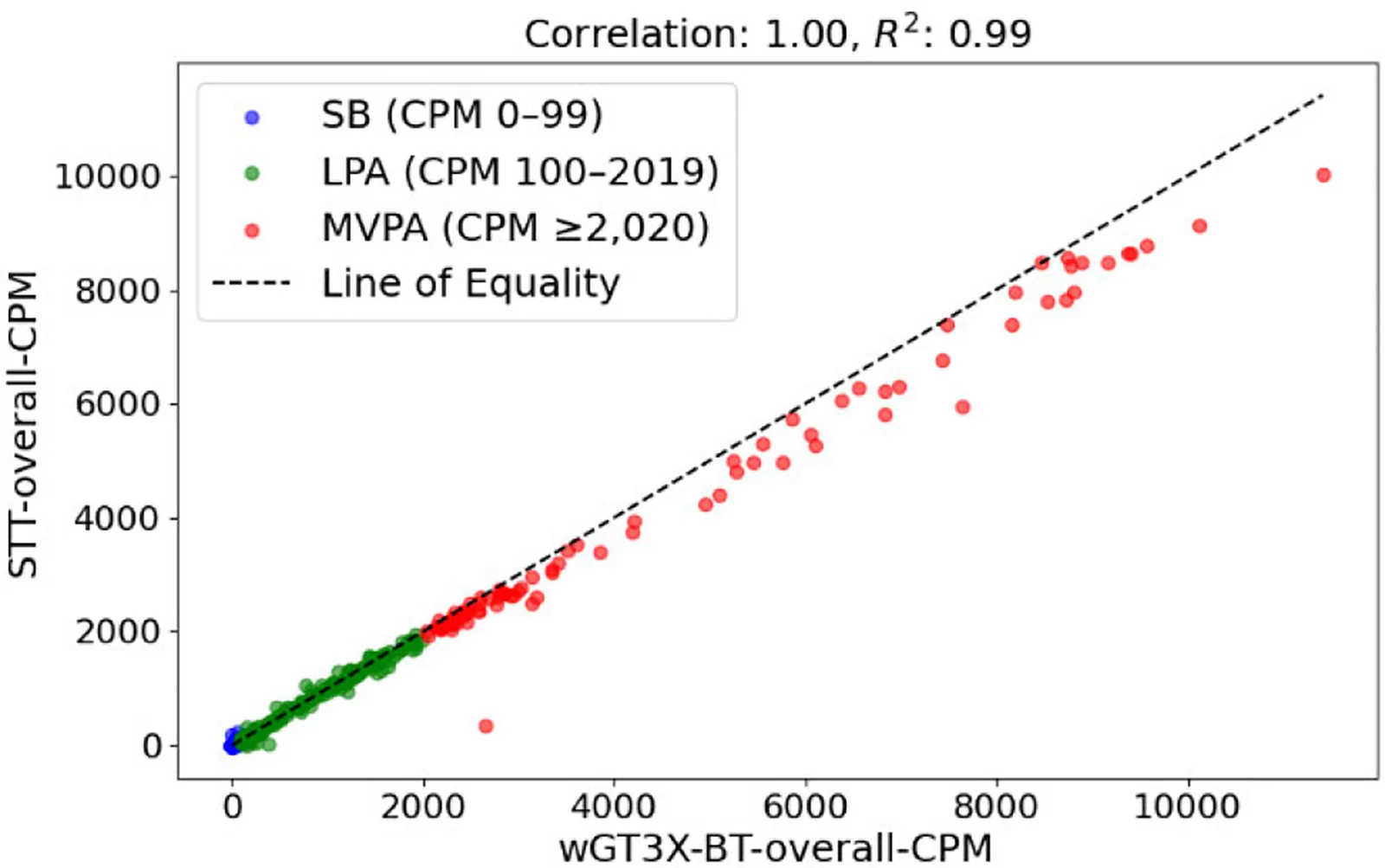
Scatter plot of STT vs. wGT3X-BT CPM for sedentary behavior (SB), light physical activity (LPA), and moderate-to-vigorous physical activity (MVPA).

**FIGURE 7.**
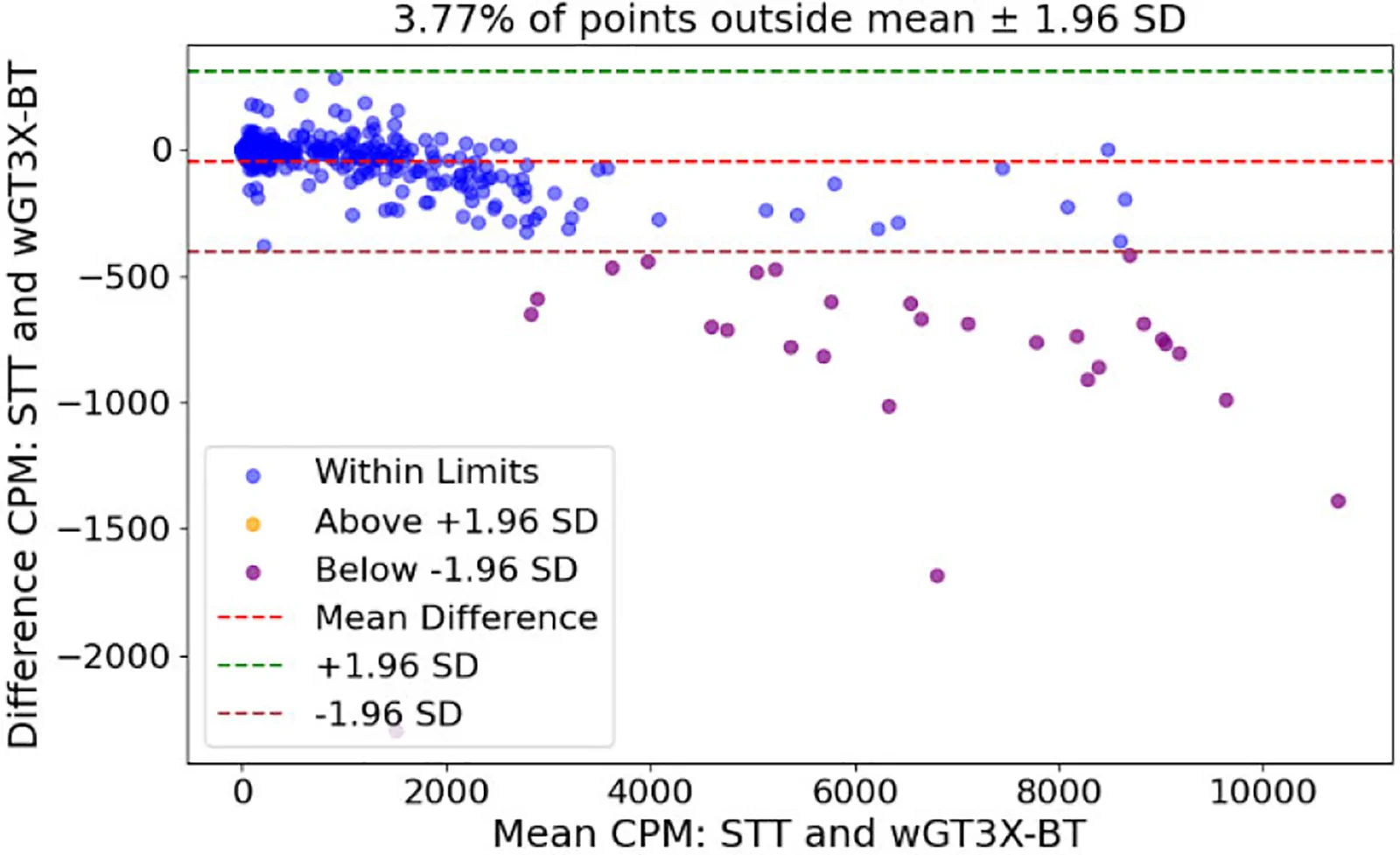
Bland-Altman plot of STT vs wGT3X-BT CPM for sedentary behavior, light physical activity, and moderate-to-vigorous physical activity.

The scatter and Bland-Altman plots (Fig. 6 and 7) demonstrate strong agreement between STT and wGT3X-BT CPM values. Table 1 presents quantitative metrics, including average deviation, root mean square error, Pearson correlation, and Lin’s concordance correlation coefficient. Lin’s concordance correlation coefficients indicated lower agreement for sedentary behavior (0.84) than for light physical activity (0.99) and moderate-to-vigorous physical activity (0.98). For sedentary behavior and light physical activity, STT could generate both under- and over-estimated activity counts compared to wGT3X-BT. On the other hand, for moderate-to-vigorous physical activity, STT systematically underestimated activity counts probably because of its lower dynamic range (±2g for STT versus ±8g for wGT3X-BT). The root mean square errors were 17.65 CPM, 69.69 CPM, and 541.45 CPM for sedentary behavior, light physical activity, and moderate-to-vigorous physical activity, respectively. However, because root mean square error is scale-dependent, its increase across activity categories should be interpreted relative to the corresponding CPM ranges and does not alone indicate lower agreement. Taken together, the strong Pearson correlations and Lin’s concordance coefficients, together with the Bland–Altman plots, indicate that STT-derived CPM values are reliable and closely comparable to wGT3X-BT measurements.

### B. VALIDATION OF SCREEN IMAGE DETECTION AND SCM CALCULATION

To assess the accuracy of the YOLOv5l6 model on the children’s study, we created a test dataset of 8,490 annotated screen and non-screen images (representing approximate 23 hours 35 minutes of data). The YOLOv5l6 model’s performance was evaluated at both the screen image detection and SCM. For the selected confidence threshold, metrics including accuracy, precision, recall, F1-Score, specificity, negative predictive value, area under the curve, and false positive rate were also calculated [53].

**FIGURE 8.**
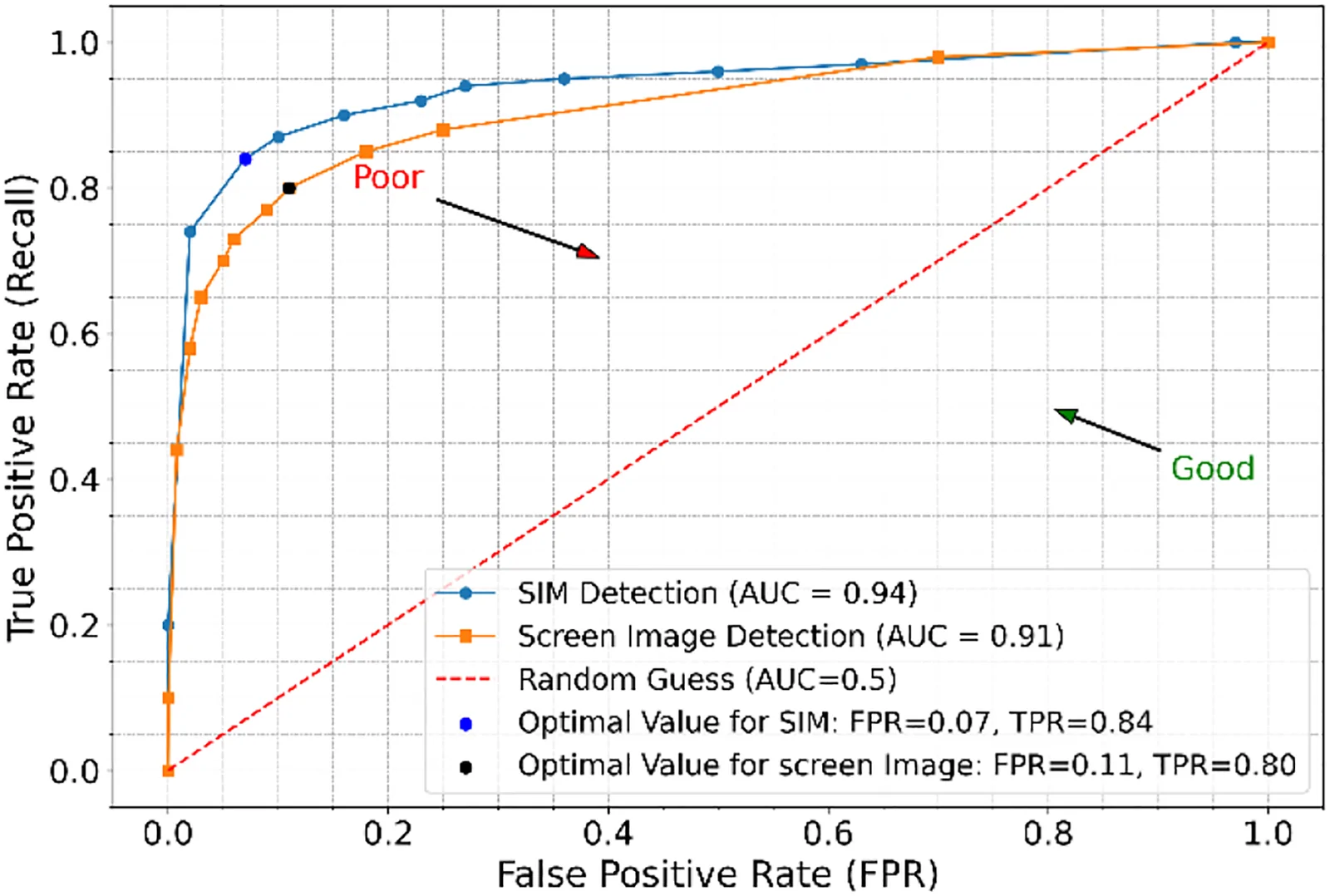
ROC curves for screen image and screen interaction detection.

Fig. 8 presents the ROC curves for screen image and SIM detection. We used a confidence threshold of 0.7 for SIM detection, despite single-image detection achieving higher accuracy at a threshold of 0.2, as the higher threshold minimized false positives in SIM detection. At a confidence threshold of 0.7, the model achieved an area under the curve of 0.94 (where 1.0 indicates a perfect model). Table 2 summarizes the YOLOv5l6 model’s performance metrics for screen image and SIM at a confidence threshold 0.7. The model achieved a false positive rate of 0.07 for SIM detection, with accuracy, precision, recall, and F1-score of 0.89, 0.92, 0.84, and 0.88, respectively. Our single-view model’s F1-score of 0.88 exceeds the 0.81 reported by [54] and approaches the 95.5% accuracy achieved by the larger multi-view VLM model in [35], despite using a substantially smaller architecture and only one view.

### C. VALIDATION OF WEAR-TIME COMPLIANCE DETECTION

The performance of the threshold-based wear-time compliance detection algorithm was evaluated against the manually annotated ground truth. When applied to the full dataset, the selected threshold yielded 198.95 h of true-positive detections, 60.27 h of true-negative detections, 18.10 h of false-positive detections, and 2.00 h of false-negative detections. These results corresponded to a precision of 0.92, recall of 0.99, F1 score of 0.95, and accuracy of 0.93; the definitions of these accuracy metrics are provided in [53]. False-negative detections included only 162 screen images, corresponding to approximately 27 min of missed screen-use exposure. After threshold-based detection, only algorithm-detected normal-wear intervals were retained for further analysis. In total, 217.05 h of algorithm-detected normal-wear data were retained, whereas algorithm-detected non-wear intervals and their corresponding images were excluded from analyses.

### D. ST EFFECT ON PA INTENSITY

The effect of screen time on PA intensity is examined in two analytical components: (1) the moment-to-moment relationship between screen detection frequency and PA intensity, and (2) the association between longer consecutive minutes of screen-exposure bouts and suppression of PA. Descriptive statistics and multilevel models were used to address each component. The following subsections present the results for each analysis.

#### 1) THE MOMENT-TO-MOMENT RELATIONSHIP BETWEEN SCREEN DETECTION FREQUENCY AND INTENSITY OF PA

**FIGURE 9.**
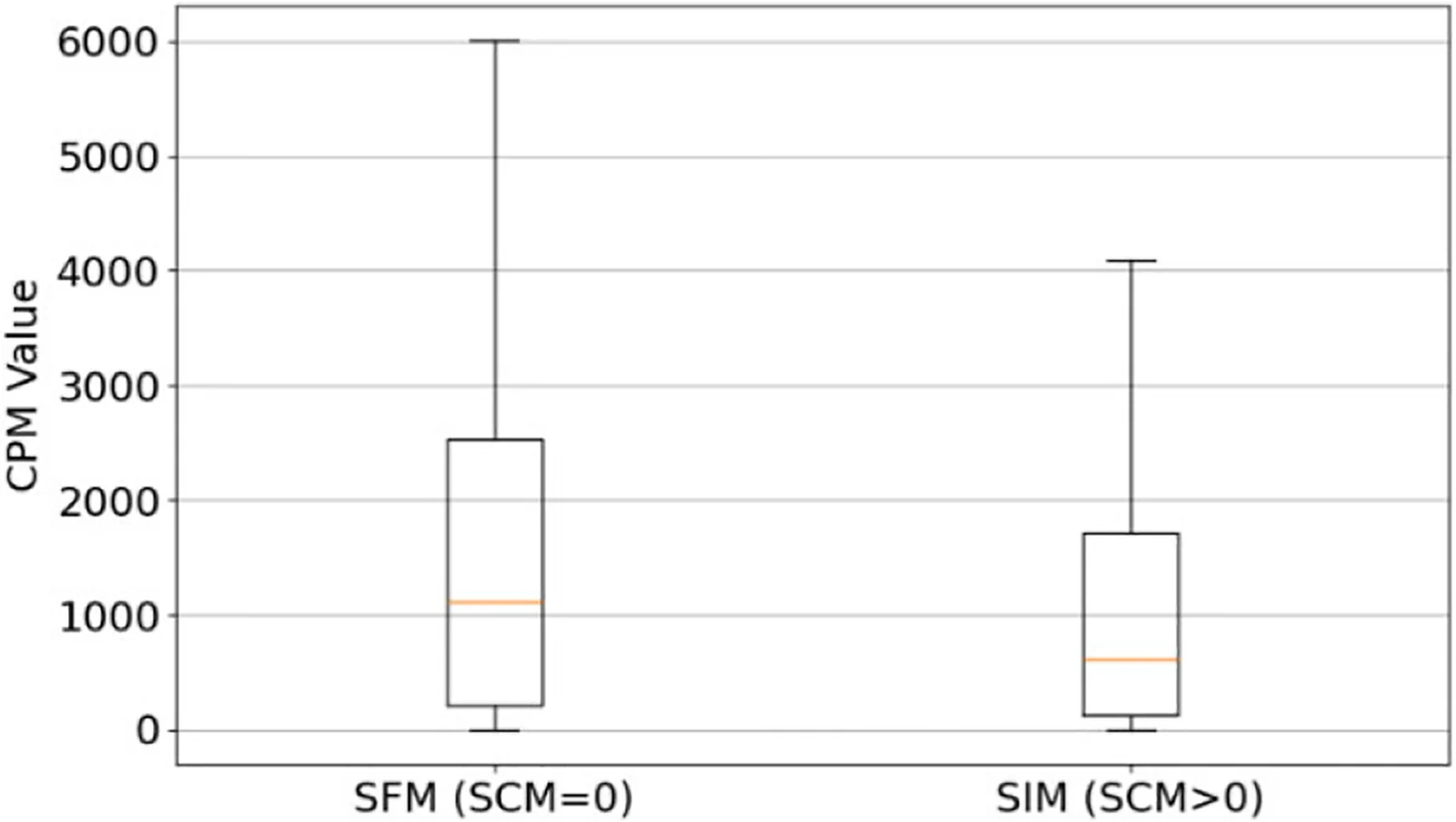
Whisker plot of CPM for SFM and SIM combining all children participants’ data.

**FIGURE 10.**
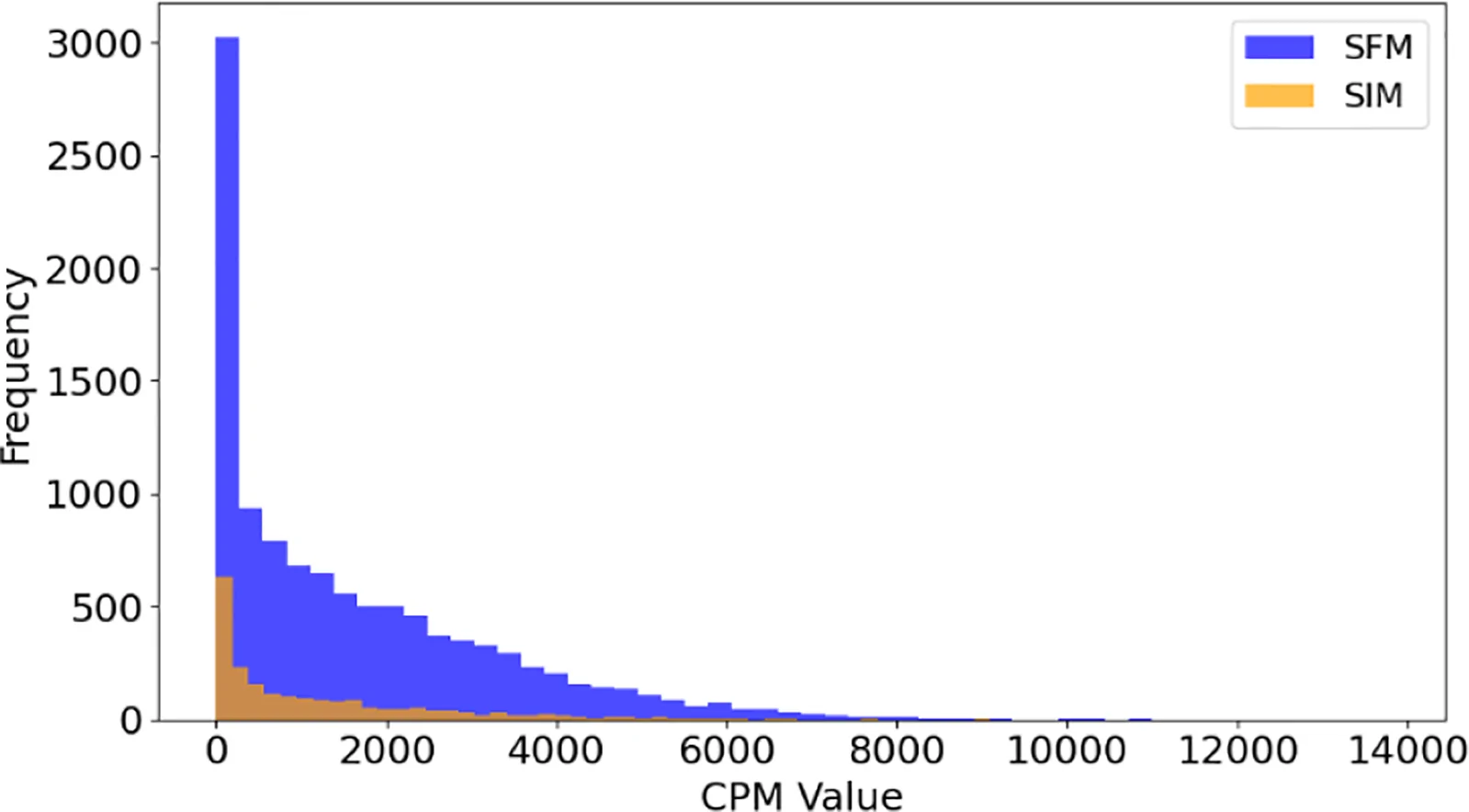
CPM distributions for SFM and SIM.

Fig. 9 and 10 display the whisker plot and distribution of CPM values for all child participants. Fig. 9 shows that SFM exhibits higher CPM than SIM, and the distribution further confirms this observation. The average CPM during SFM was 1628 ± 1709, compared with 1181 ± 1425 during SIM. The median CPM also followed this trend, decreasing from 1113 CPM in SFM to 609 CPM in SIM. Although CPM values showed substantial variability in both SFM and SIM, the consistent differences in both mean and median indicate that screen interaction is associated with lower CPM and reduced PA intensity.

The hierarchical structure of the data necessitated multi-level modeling to determine whether minutes with greater screen exposure are associated with lower physical activity (i.e., lower CPM). We estimated a three-level model in which minutes (level 1) were nested within days (level 2), and days were nested within children (level 3). The day level was included because 8 of the 25 children contributed two days of data, and this structure accounts for correlations among minutes from the same day. For each child, minute-level SCM values were centered around the child- and day-specific mean SCM. This centering isolates within-person and within-day variation, addressing the question: “On a given day, during minutes when a child experiences more screen exposure than their own daily average, does their physical activity tend to be lower?” Time of day (morning, afternoon, evening) was included as a covariate, defined consistently for all participants as: morning = before 11:00 a.m., afternoon = 11:00 a.m.–5:00 p.m., and evening = after 5:00 p.m. Random effects included random intercept and random effects of SCM at level 2 and level 3. The negative binomial distribution and the log link were specified. The model was estimated using SAS macro HPGLIMMIX in SAS/STAT. The result suggested that, in a typical day, in the minute that a typical child had higher screen counts, s/he tended to have lower activity counts (b = −.228, p <.001, exp(b) = .796). As screen count increases by 1, activity count is expected to change by a factor of 0.796 (i.e., decrease by 20.4%).

#### 2) CORRELATION OF LONGER CONSECUTIVE MINUTES OF SCREEN EXPOSURE BOUTS TO PA SUPPRESSION

**FIGURE 11.**
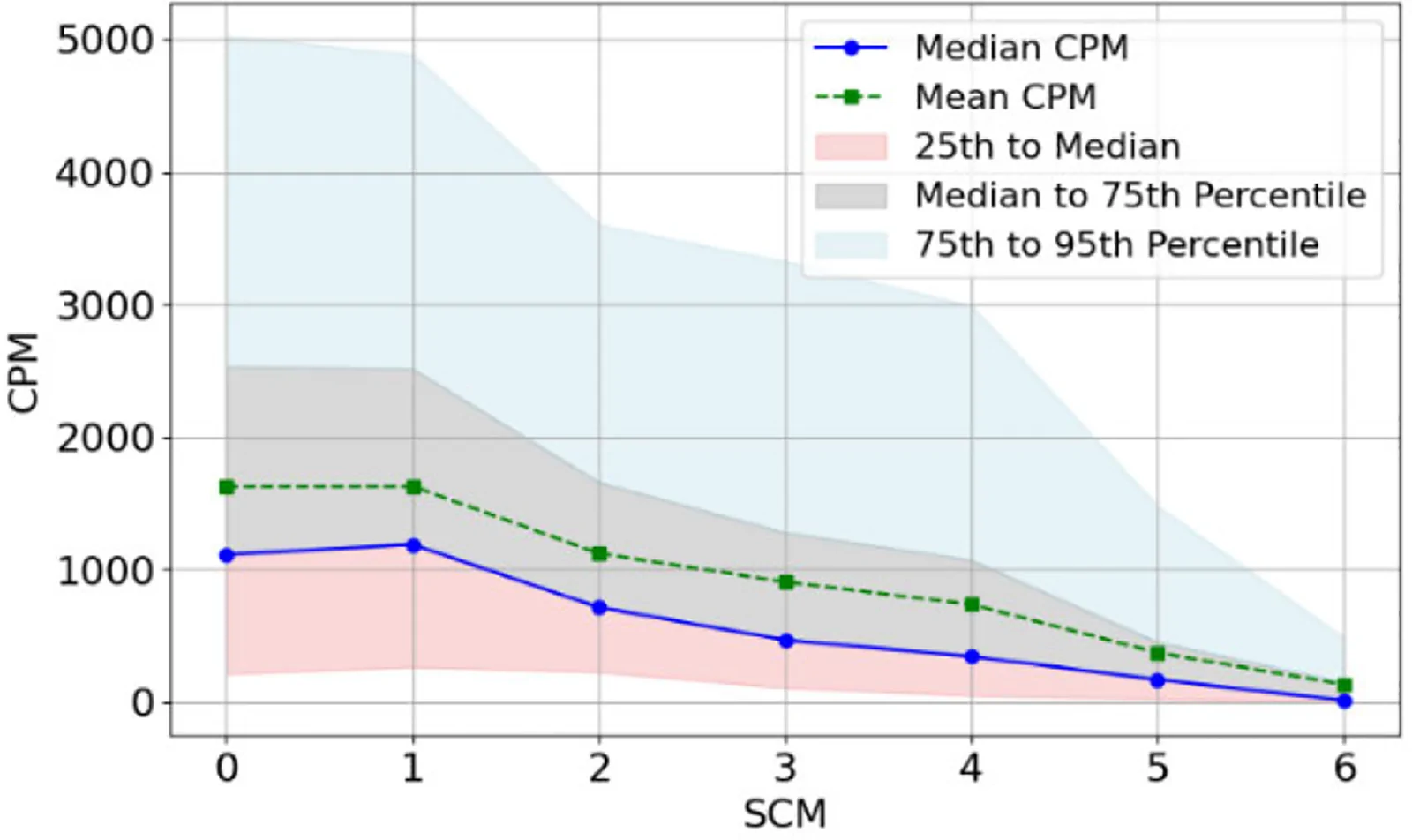
Mean and median CPM as a function of SCM. Higher SCM values were associated with progressively lower CPM, indicating that greater screen-detection frequency was associated with lower physical activity intensity.

Fig. 11 shows the mean and median CPM for different SCM values, with CPM decreasing as SCM increases. Table 3 presents the descriptive statistics for CPM across SCM values. We observed that the mean CPM is 1628 when SCM is zero, which is similar for SCM 0 or 1. This can be attributed to a few factors: first, the YOLOv5l6 model, while effective, is not perfect and may introduce some false positives and negatives. Additionally, there are instances when children may look at the screen without actively interacting with it, leading to similar CPM values for SCM of zero and one. As screen interaction (i.e., SCM) increases, indicating more active engagement with screens, the intensity of physical activity (i.e., CPM) decreases, dropping to a mean CPM of 132 when SCM reaches its maximum value of 6.

**FIGURE 12.**
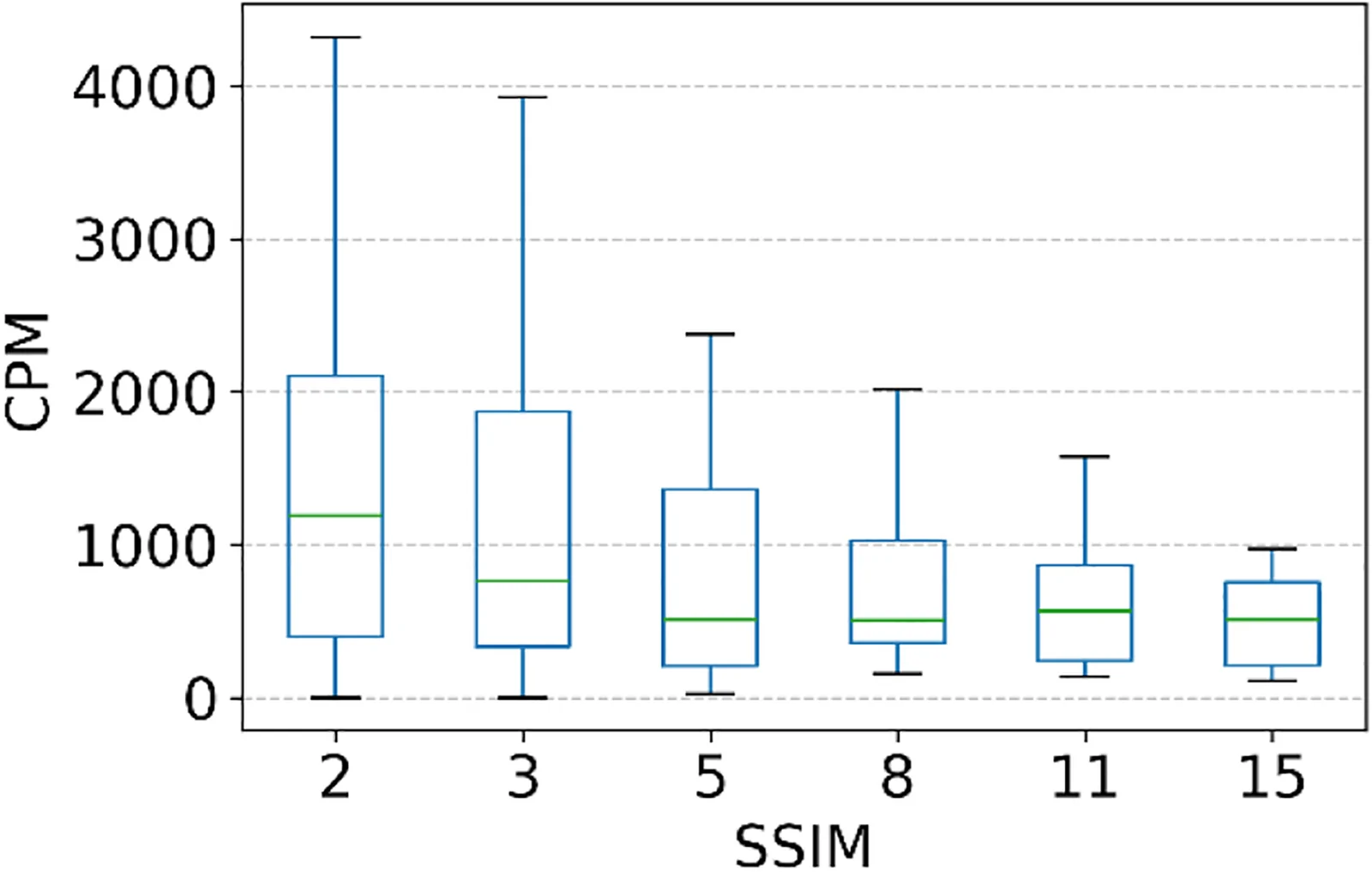
Whisker plot of CPM across SSIM.

Next, we plotted a whisker plot showing the distribution of CPM values for SSIM (Fig. 12). We found that as SSIM increases, CPM decreases. The mean CPM was 1629 (Table 3) for SCM of 1, while it dropped to 732 mean CPM for 11 SSIM (Table 4). It further reduces to 693 CPM when 15 minutes or more of SSIM. Additionally, we observed that the mean CPM remains similar for 8 and 11 SSIM before dropping at 15 minutes. We can draw two conclusions from Fig. 11, 12, and descriptive statistics from Tables 3 and 4. First, higher SCM values are associated with lower CPM, indicating that more intense screen interaction is linked to reduced physical activity intensity. Second, higher SSIM corresponds to lower CPM values, suggesting that prolonged consistent screen interaction decreases the intensity of physical activity.

To statistically examine whether longer SSIM (i.e., longer consecutive minutes of screen-exposure bouts) was associated with lower average physical activity, we constructed non-overlapping SSIM bouts using the minute-level SCM data. For each bout, minute-level CPM values were averaged to obtain the mean CPM. We then fit a three-level model with SSIM bouts at level 1, nested within days (level 2), and days nested within children (level 3). SSIM values were centered at the child- and day-specific mean SSIM to isolate within-person, within-day associations. The model included random intercepts and random slopes for SSIM at levels 2 and 3 and was estimated using a normal distribution with an identity link. Although SSIM is defined as ≥2 consecutive minutes with SCM > 0, we also conducted a secondary analysis including bouts ≥1 minute, since some children may not exhibit SSIM ≥2 if every screen-exposed minute is followed by a screen-free minute. There were 383 SSIMs ≥ 2 minutes, totaling 1624 minutes, averaging 4.240 minutes (min = 2 minutes, median = 3 minutes, max = 29 minutes). Results suggested that, for a typical child on a typical day, CPM is lower during a longer SSIM (b = −46.677, p = .005). For a 1-minute increase in SSIM, mean CPM tends to decrease by 46.677 units. In the secondary analysis, we included SSIMs that were ≥ 1 minute. There were 877 such SSIMs, totaling 2118 minutes, averaging 2.415 minutes (min = 1 minute, median = 1 minute, max = 29 minutes). Result points to the same conclusion, suggesting that, for a typical child in a typical day, mean CPM tended to be lower during a longer SSIM (b = −75.686, p <.001).

## IV. CONCLUSION

In this study, we present a first-of-its-kind approach for the quantitative analysis of the impact of media screen exposure on physical-activity counts in preschool children aged 3–5 years. Using YOLOv5l6 for screen image detection and activity count calculations derived from the STT accelerometer via the Actigraph Python package, we developed and validated a fully automated system for measuring screen time exposure and physical activity. Moreover, we considered a wide variety of media screens in our analysis. Our findings reveal a negative correlation between screen presence and physical activity counts, with higher screen image presence per minute and prolonged screen interactions leading to reduced activity intensity among preschoolers. The statistical significance test also strongly supports the conclusion. Moreover, the study was conducted in a free-living environment, offering a more realistic and unbiased analysis of media screen effects.

This work has several limitations that will guide future research. First, the performance of the system depends on the accuracy of the screen-detection model. Although the YOLOv5l6 model performed well, false positives and false negatives remain possible and may influence the precision of screen-exposure estimates. Screen detection performance may be affected by screen distance, size, brightness, color, apparent resolution, and whether the screen is partially blocked from view in egocentric images. Moreover, the performance of the wear-compliance detection method may also affect the overall output, since misclassification of wear and non-wear periods could lead to inclusion of invalid data or exclusion of valid data. Future work will focus on further improving model accuracy and robustness to ensure more reliable performance across diverse contexts.

Second, this study did not differentiate among types of screens or modes of engagement. Different screen activities—such as being read to on a tablet, passively watching television, or interacting with an active video-game console—may have different effects on movement patterns. In addition, the current framework calculates the digital markers SCM and SSIM based on visible powered-on screens in egocentric images, rather than direct confirmation that the child was actively looking at the screen. Therefore, if a screen remains on without active child engagement, this may introduce bias into the SCM- and SSIM-based relationship between screen exposure and physical activity intensity. Future research will examine screen type and interaction mode to better understand how specific forms of digital media influence physical activity. Future studies should also examine whether screen engagement is associated with physical activity beyond periods of detected screen exposure, including whether more sedentary screen use is related to lower activity during subsequent screen-free periods. Real-time screen detection is feasible [55] and could be integrated into this method for future intervention work, alongside privacy-preserving approaches [56]. Future studies could also explore eye-tracking-based methods to identify active screen viewing and reduce bias in SCM and SSIM caused by screen presence without actual child engagement.

Third, the wear-compliance classification introduces additional bias. Non-wear time was simplified to non-wear-stationary only, as non-compliant-wear and non-wear-carried instances were minimal in this study but may be more prevalent in larger cohorts. For example, camera obstruction by clothing during non-compliant wear could lead to missed screen detections and bias SCM and SSIM estimates. Additionally, false-positive and false-negative detections from the threshold-based algorithm may further affect the statistical associations. Future studies should address these classification limitations before conducting larger cohort-based analyses.

Finally, this study should be viewed as a proof-of-concept evaluation of the proposed digital markers for objective quantitative assessment, rather than as an attempt to define acceptable screen-time thresholds for children. Because data were collected from a relatively small sample of 25 preschool-age participants, each monitored for one to two days, the findings have limited generalizability and may not fully capture variation across household routines, socio-economic backgrounds, and cultural practices. Although our previous study [35] has indicated high comfortableness and acceptance for regular use, we also did not directly examine whether wearing the device affected children’s behavior, so some influence of device awareness may still be present. In addition, we did not assess sleep quality, fatigue, or day-to-day variability, which may influence children’s physical activity and screen engagement. Future studies with larger and more diverse cohorts, longer monitoring periods, and direct assessment of potential device-related behavioral reactivity, sleep quality, fatigue, and daily variation could use these digital markers to better evaluate screen-time thresholds and strengthen the evidence base.

Despite these limitations, our research provides valuable insights into the relationship between media screen exposure and preschool children’s physical activity patterns. The primary contribution of this study is the development and proof-of-concept application of a nonintrusive wearable framework that combines egocentric screen detection with accelerometry and introduces SCM and SSIM as quantitative digital markers of screen engagement under free-living conditions. Using these objective markers, we demonstrated that greater screen interaction was associated with lower physical activity in preschool-aged children, extending previous findings that have largely relied on parent reports or device-specific monitoring. This provides additional evidence that greater screen exposure may coincide with less active play. This is an important public health issue, as habits formed in early childhood may influence later obesity risk, motor development, and health behaviors. These findings help guide future research and provide helpful information for epidemiologists, pediatricians, and behavioral scientists studying the effects of media screens on children’s health and behavior.

## Figures and Tables

**TABLE 1.** Numerical comparison of STT CPM considering wGT3X-BT as ground truth for SB, LPA and MVPA.

Performance metrics	SB (CPM 0–99)	LPA (CPM 100–2019)	MVPA (CPM≥2020)
Average deviation of CPM (mean±SD)	0.05±17.65	−16.4±67.74	−375.0±390.56
Mean absolute error	6.6	42.3	376.44
Root mean square error	17.65	69.69	541.45
Pearson Correlation	0.84	0.99	0.99
Lin’s concordance correlation coefficient.	0.84	0.99	0.98

Note: SB = sedentary behavior; LPA = light physical activity; MVPA = moderate-to-vigorous physical activity.

**TABLE 2.** Performance of the YOLOv5l6 model for Screen Image Detection (SID) and SIM detection in the Children’s dataset.

	CT	A	P	R	F-1	S	FPR	NPV
SID	0.7	0.86	0.92	0.58	0.71	0.98	0.02	0.85
SIM	0.7	0.89	0.92	0.84	0.88	0.93	0.07	0.87

Note: CT = confidence threshold; A = accuracy; P = precision; R = recall; S = specificity; F1 = F1-score; NPV = negative predictive value; FPR = false positive rate.

**TABLE 3.** Statistical summary of CPM for different SCM.

Descriptive statistics	SCM						
	0	1	2	3	4	5	6
Mean CPM	1628	1629	1122	907	737	373	132
STD of CPM	1709	1644	1230	1143	962	507	312
Median	1113	1189	715	469	344	170	13

**TABLE 4.** Summary statistics of CPM for SCM = 1 and different SSIM.

CPM	SCM	SSIM					
	1	2	3	5	8	11	15
Mean	1629	1421	1165	948	766	732	693
STD	1644	1301	1139	1065	600	691	754
Median	1189	1191	763	508	502	564	508
